# Autonomy support encourages use of more-affected arm post-stroke

**DOI:** 10.1186/s12984-023-01238-0

**Published:** 2023-09-07

**Authors:** Sujin Kim, Yumi Shin, Yeonwoo Jeong, Seungyoung Na, Cheol E. Han

**Affiliations:** 1 Department of Physical Therapy, Jeonju University, Jeonju, South Korea; 2 Department of Rehabilitative and Assistive Technology, National Rehabilitation Center, Seoul, South Korea; 3 Department of Rehabilitation and Medicine, Ongoul Rehabilitation Hospital, Jeonju, South Korea; 4 Department of Electronics and Information Engineering, Korea University, Sejong, South Korea; 5 Interdisciplinary Graduate Program for Artificial Intelligence Smart Convergence Technology, Korea University, Sejong, South Korea

**Keywords:** Choice behavior, Hemiparesis, Motivation, Self-efficacy, Upper extremity

## Abstract

**Background:**

Autonomy support, which involves providing individuals the ability to control their own behavior, is associated with improved motor control and learning in various populations in clinical and non-clinical settings. This study aimed to investigate whether autonomy support combined with an information technology (IT) device facilitated success in using the more-affected arm during training in individuals with stroke. Consequently, we examined whether increased success influenced the use of the more-affected arm in mild to moderate subacute to chronic stroke survivors.

**Methods:**

Twenty-six participants with stroke were assigned to the autonomy support or control groups. Over a 5-week period, training and test sessions were conducted using the Individualized Motivation Enhancement System (IMES), a device developed specifically for this study. In the autonomy support group, participants were able to adjust the task difficulty parameter, which controlled the time limit for reaching targets. The control group did not receive this option. The evaluation of the more-affected arm's use, performance, and impairment was conducted through clinical tests and the IMES. These data were then analyzed using mixed-effect models.

**Results:**

In the IMES test, both groups showed a significant improvement in performance (p < 0.0001) after the training period, without any significant intergroup differences (p > 0.05). However only the autonomy support group demonstrated a significant increase in the use of the more-affected arm following the training (p < 0.001). Additionally, during the training period, the autonomy support group showed a significant increase in successful experiences with using the more-affected arm (p < 0.0001), while the control group did not exhibit the same level of improvement (p > 0.05). Also, in the autonomy support group, the increase in the use of the more-affected arm was associated with the increase in the successful experience significantly (p = 0.007).

**Conclusions:**

Combining autonomy support with an IT device is a practical approach for enhancing performance and promoting the use of the more-affected upper extremity post-stroke. Autonomy support facilitates the successful use of the more-affected arm, thereby increasing awareness of the training goal of maximizing its use.

**Trial registration:**

The study was registered retrospectively with the Clinical Research Information Service (KCT0008117; January 13, 2023; https://cris.nih.go.kr/cris/search/detailSearch.do/23875).

## Background

Functional improvements obtained through rehabilitation often do not increase the use of the more-affected arm in real-world situations [1, 2]. Multiple clinical trials on constraint-induced movement therapy [3, 4], task-oriented movement therapy [5, 6], and high-dose training [7, 8] have aimed to overcome this discrepancy between function and use of the upper extremity (UE) by increasing the use of the more-affected arm. In the same context, the factors influencing the use of the more-affected arm have been explored, and attempts have been made to incorporate them into treatment approaches to improve their therapeutic effects. These factors include cost (e.g., biomechanical effort), reward (e.g., task success), side of stroke, and hand preference before stroke, which affect the decision of arm choice [9–11]. Recently, socio-cognitive behavioral factors such as autonomy support, self-efficacy, and attention/arousal have been gaining attention for their potential impact on the spontaneous use and recovery of the UE post-stroke [12–16].

Autonomy support facilitates the basic psychological need of individuals to control their own behaviors [17, 18]. Known to facilitate motor control and learning in clinical and non-clinical populations [19, 20], it is now among the principal concepts of patient-centered intervention programs. For instance, such intervention programs encourage patients to actively participate in planning and selecting task order and difficulty [6]. Autonomy support has also been found to be associated with self-efficacy. [18]. Self-efficacy, one’s belief in their capacity to complete a certain task [21], is an important factor affecting use of the more-affected arm [12, 22]. While such socio-cognitive behavioral factors have increasingly emerged in stroke rehabilitation, it remains necessary to investigate their effects on the dynamics of recovery.

Here we examined the effect of autonomy support on the use of the more-affected arm post-stroke. Since previous studies indicated that autonomy support led to better performance [18, 19, 23], we hypothesized that autonomy support would boost task success with the more-affected arm, and consequently, the accumulated success would facilitate the use of that arm. This aligns with the context of the previous research that successful performance with the more-affected arm, especially combined with high repetitions, can reinforce its spontaneous use [4, 24–26]. Thus, this study included simple high-dose repetitive reaching movements with autonomy support wherein the patients actively participated in selecting task difficulty. This training approach might promise patients with stroke a greater chance of successful outcomes. Concurrently, to maximize the learning effect on spontaneous use of the more-affected arm, we applied “choice training,” which allowed participants to choose which arm to use.

A distinctive feature of our study was the utilization of a novel information technology (IT) device, which allowed patients to adjust their task difficulty, thereby facilitating autonomy support. A previous study on UE training via IT systems was successful in attaining increased use of the left non-dominant arm in healthy participants [27], while visual augmentation providing a more successful experience improved the use of the more-affected arm in stroke survivors [28]. However, these systems automatically changed the task difficulty based on the performance, so they did not monitor dynamic changes in patients’ behaviors during training sessions, such as the task difficulty selection and changes in motivation variables. Our novel system overcomes these limitations by allowing the active involvement of participants in changing task difficulty or training schedules during training.

This study aimed to investigate the effects of autonomy support on the ability of the participants with stroke successfully use their more-affected arm both during and after training. In addition, we examined the dynamic changes in self-efficacy and association with the use of the more-affected arm.

## Methods

### Participants

**Fig. 1:**
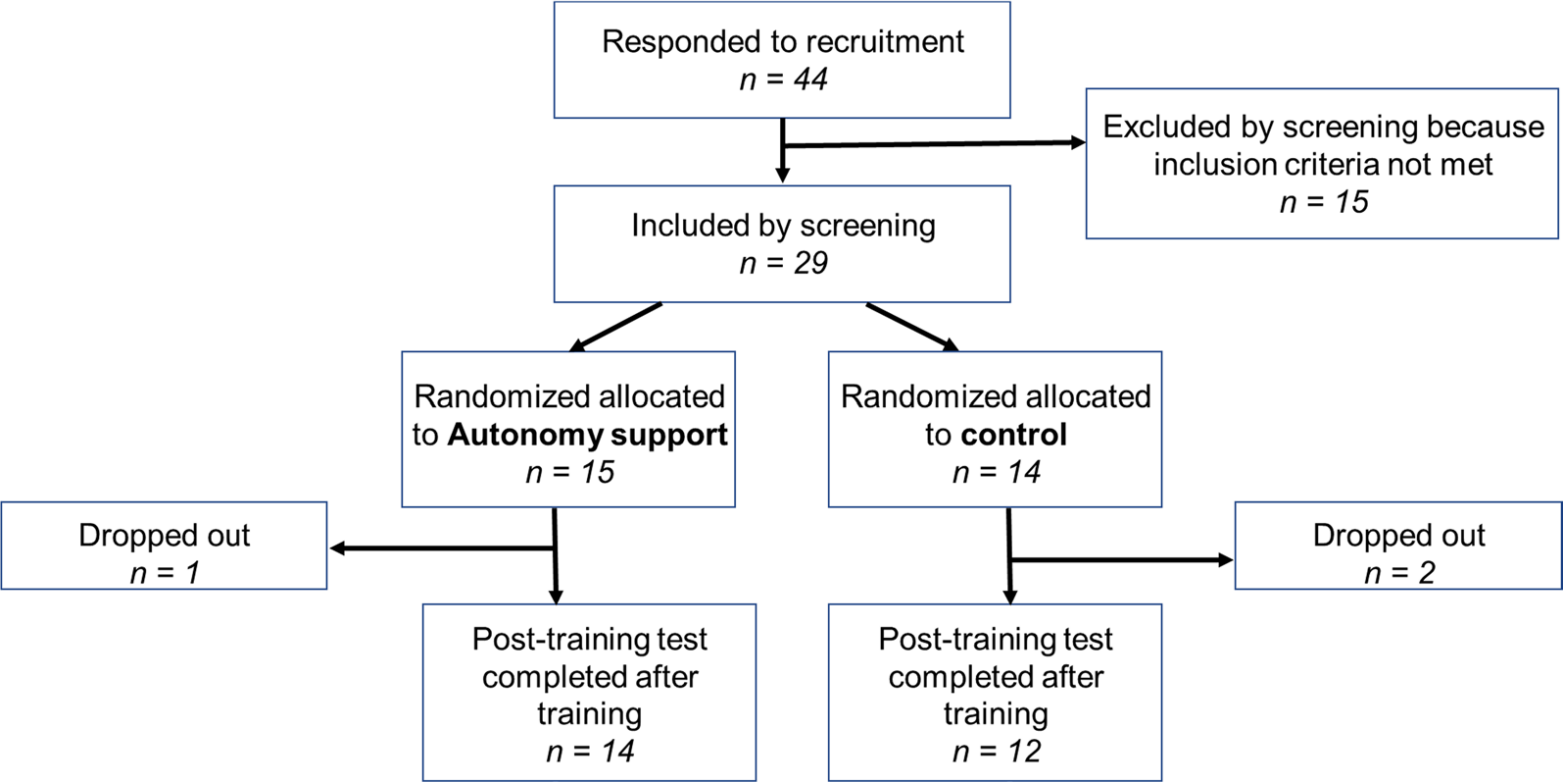
CONSORT (Consolidated Standards of Reporting Trials) diagram for the present study

Twenty-nine patients with subacute to chronic stroke were enrolled in this study (Fig. 1) and allocated to autonomy support and control groups. To ascertain the necessary sample size for observing the influence of autonomy support on the use of the more-affected arm (as measured by IT system we developed), a simulation was conducted. We carried out a priori power analysis using the MixedPower package in R, which is particularly appropriate for power analysis in mixed-effect models [29]. This package employs simulation to estimate power and we specified initial parameters based on our prior dataset [30]. The simulation indicated that a sample size of twelve participants would suffice to achieve at least 80% power at an alpha level of 0.05. However, to compensate for potential participant dropouts, we decided to recruit extra participants, thus establishing a final sample size of 15 for each group. After dropouts, a total of twenty-six participants with stroke completed the whole training and test sessions.

Table 1 presents the participants demographics. All participants were right-handed, had mild to moderate impairment in the UE, and had the motor capability to complete the task using their more-affected arms. The inclusion criteria were as follows: (1) first-ever ischemic or hemorrhagic stroke in the subacute or chronic stage (at least 1 month after stroke onset); (2) movement difficulties in the upper limb (Fugl-Meyer Assessment [FMA] score ≥ 19)[8, 31]; (3) no communication or intellectual problems (Mini Mental State Examination score ≥ 24); (4) right-handedness (Edinburgh handedness score ≥ 70%); and (5) ability to reach all targets displayed on a touch screen used in the experiment. The exclusion criteria were: (1) multiple stroke attacks or having undergone orthopedic surgery on the UE; and (2) visuomotor neglect as measured by Albert’s test [32]. The participants were randomized into the autonomy support and control groups. Those in the former completed a training schedule to learn the use of the more-affected arm, while those in the latter did not. This study was approved by the Institute of Research Board of Jeonju University (IRB-190414-HR-2019-0409). All participants read and signed a written consent form before starting the experiment.

**Table 1:** Demographic information for control and autonomy support groups (N = 26)

Variable		Control (n = 12)	Autonomy support (n = 14)	p-value
Age		62.17 ± 5.84	61.14 ± 12.93	0.793
Stroke types				
Hemorrhage		7	7	
Infarction		5	7	
Affected side				
Left		5	4	
Right		7	10	
Gender				
Female		2	3	
Male		9	11	
Stroke month		29.83 ± 28.26	56.43 ± 48.56	0.08
FMA-UE^a^		47 ± 12.64	42 ± 17.28	0.643
MMSE^b^		28.58 ± 1.78	29.36 ± 1.01	0.302
EHI^c^		92.50 ± 10.55	97.14 ± 7.26	0.156

^a^FMA-UE, Fugl-Meyer Assessment for Upper Extremity

^b^MMSE-K, Mini Mental State Examination-Korean

^c^EHI, Edinburgh Handedness Inventory

### Individualized motivational enhancement system

**Fig. 2:**
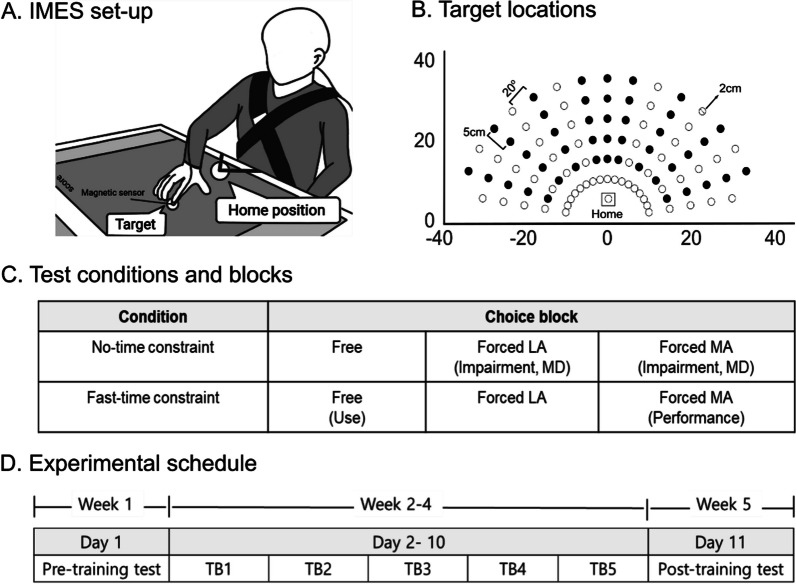
**A** The 55-inch touchscreen was placed on the table to adjust the height. The participant sat on the chair, and the trunk was constrained with a belt to prevent compensational movement. All participants were able to reach the farthest target by adjusting the table height. **B** One hundred target locations were predefined for the training session. Forty-five targets (filled circles) were used for the test session. **C** IMES test conditions and blocks. IMES had a test session with and without a time limit. Each condition had free- and forced-choice blocks, and the forced-choice block started from the less-affected (LA) arm to the more-affected (MA) arm. Movement duration (MD) for each arm was measured during these blocks. **D** Experiment schedule. The first and last weeks were the pre- and post-training tests, and the middle three weeks were training sessions conducted three times per week. TB, training block

Here we developed and used a new training system called the Individualized Motivation Enhancement System (IMES), which changes task difficulty by varying the time limit for the reaching movements. The IMES was inspired by our previous system, the Bilateral Arm Reaching Test (BART) [30, 33]. The IMES comprises a 55″ touch screen that provides targets and collects data; a main computer that controls the overall programs; and a height-controllable desk that adjusts the screen to the height of individual participants (Fig. 2A). Our system provides game-based testing and individualized training sessions that require participants to catch targets on the 2D touch screen by moving their more- or less-affected arms (see “IMES: test and individualized training sessions” below for details).

At the beginning of the sessions, the participants were asked to place their index fingers in the home position, which was set 20 cm from the xiphoid process of the trunk. The target then appeared in one of the 100 predefined locations (17 angles between 10° and 170° at 20-degree intervals, and six different distances between 10 and 30 cm at 5-cm intervals; Fig. 2B). The maximum distance of the targets was 50 cm from the subject. The participants were instructed to “catch the target as fast as possible before the targets disappear and then return to the home position.” Each target appeared and disappeared within a predefined time limit. A movement was counted as successful only when the participants successfully caught the target within the set time limit. If the participants successfully caught the targets, they earned game points, which appeared in the top-right corner of the screen for motivational purposes. The total number of targets and time limits varied across the test and training sessions as explained below.

### IMES: test session

During the IMES test session, impairment, performance, and use of the more-affected arm were measured (Fig. 2C). Forty-five of the 100 targets were used in the test session (Fig. 2B). There were two different conditions: no-time constraint and a fast-time constraint. The no-time constraint condition had no time limit, while the fast-time constraint condition had an approximately 500 ms time limit. The time limit in the fast-time constraint condition was based on that in a previous study that investigated different movement durations for targets in different locations [30, 34]. Each condition had free- and forced-choice blocks. During the free-choice block, the participants freely selected the arm they used. During the forced-choice block, the participants were asked to use only the less-affected arm and then the more-affected arm to reach all targets. In the free-choice block, each target appeared twice, resulting in a total of 90 targets. In the forced-choice block, each target appeared once, resulting in a total of 45 targets. The total number of targets that the participants successfully reached in the free- and forced-choice blocks in the fast-time constraint condition represented the more-affected arm performance and use, respectively [30, 33]. Since the participants were informed that there were no correct or wrong answers for the free-choice blocks, the spontaneous arm choice pattern (i.e., use of the more-affected arm) was measured. We also included the forced-choice block in the no-time constraint condition to measure the movement duration for each arm and used each movement duration to designate the time limit for the training session.

### IMES: individualized training session

The IMES training session was similar to the free-choice block under the fast -time constraint condition. However, the time limit to reach each target was customized based on the individual’s functional ability. As previously mentioned, the movement duration for each target was measured for each arm during the forced-choice block under the no-time constraint condition in the IMES test session. These movement durations were set as the default time limit for catching the target with a zero task difficulty parameter (TDP) during the training session. The TDP was used to modulate the task difficulty by changing the time limit for each target so the tester or the participant manipulated the TDP between -50% and + 50% at 10% intervals (± 50%, 40%, 30%, 20%,10%, 0%). If the TDP was set at + 10% for the more-affected, the time limit became 10% longer than the actual movement time of that  arm. If the TDP was negative, the time limit was shortened. The time limit differed depending on the arm used. Most patients with stroke showed slow movement times when using their more-affected arm; thus, the durations of the more- and less-affected arms were measured separately, and these values were applied during the training. For example, for the target on the left corner, the time limit was 580 ms for the more-affected arm and 350 ms for the less-affected arm. Based on these customized time limits for the reaching task, the participants freely selected the arm they used.

There were 100 targets in each training block. The participants were asked to successfully catch the targets as many times as possible. They were informed that they could choose which arm to use, but each tried to use the more-affected arm without failure during the training sessions. If they missed several targets using one arm at the beginning, the tester recommended speeding up or switching to the other arm. The participants generally completed five training blocks, each consisting of a total of 100 targets per day, unless they felt tired or experienced pain.

### Training the more-affected arm with versus without autonomy support

To investigate the effects of autonomy support on the participants’ behavior (whether they selected and successfully used the more-affected arm), we randomized the participants into the autonomy support and control groups. The participants in the autonomy support group were able to actively adjust their task difficulty, although they did not exactly recognize how the IMES modulated it. After each training block, the participants were asked whether they wanted to make the task more difficult. The time limit became faster or slower in accordance with their choices for the TDP. In contrast, participants in the control group had no opportunity to adjust to task difficulty. Instead, it was randomly selected between − 20 and + 20 of the TDP with respect to the movement duration for each training block. On the first training day (Day 2), we intentionally set the TDP as more positive than on the other days for the control group, as we thought that this would be helpful in preventing the negative failure of using the more-affected arm.

We administered social comparative verbal feedback to the autonomy support group only. This consisted of phrases such as “the active participant, like you, normally performs well at this type of game.” However, the participants in the control group received only brief plain verbal feedback, such as “good job.”

### Experimental protocol

The overall experiment consisted of 11 visits conducted over 5 weeks (Fig. 2D). Screening examinations were performed at least 1 week before the experiment. On the first day, participants who met the inclusion criteria were randomly allocated to the study groups. All participants completed the pre-training test, including the IMES test session and clinical assessments, on Day 1 in the first week. The participants performed the five blocks of the training session during the second to fourth weeks (Days 2–10), each lasting about 1 h. The training session was performed three times a week for 3 weeks. The participants were asked about their self-efficacy immediately after each training block. In the fifth week, a post-training test, identical to the pre-training test, was conducted (Day 11).

### Outcome measurements

Our principal objective in this study was to examine the use of the more-affected arm after the training with and without autonomy support. To evaluate this main outcome, we employed the IMES system and a clinical evaluation known as the Actual Amount of Use Test (AAUT). These methods allowed us to observe changes in arm use both within our game-based system and in a clinical setting. Further details about these assessments are provided in the subsequent sections. The secondary outcomes were the impairment and performance of the more-affected arm, which were also measured using the IMES system and clinical tests. To examine the factors that influenced these primary and secondary outcomes, we analyzed behavioral changes during the training period. Specifically, we investigated how the following parameters evolved from the early to late phase of training: TDP, total use, successful and failed experiences, success rate and perseverance of the more-affected arm. We also measured self-efficacy as a motivational variable.

#### Measuring use of more affected arm

We measured the use of the more-affected arm throughout the training and test sessions using the IMES. The participants’ selected arm during the training is described below(see "Effeects of autonomy support on behavior changes"). In the pre- and post-training tests, the IMES measured the use of the more-affected arm by counting the total number of successful reaching with the more-affected arm during the free-choice block in the fast-time constraint condition (see “IMES: test session” for more details) [30, 33]. To express this measurement as a percentage, the count was divided by the total number of targets (90 targets) in this free-choice block.

A clinical test, the AAUT, was also used to evaluate use. The AAUT is a covert laboratory-based test in which participants perform daily activities using the UE (e.g., writing or turning a photo album page) according to predefined scenarios. Owing to the nature of the AAUT setup, the participants were unaware that they had been tested, which allowed us to measure the actual use of the more-affected arm [1, 35]. The tester recorded the entire AAUT process, and a blinded evaluator scored the movements based on their quality of movement (AAUT-QOM) by watching the video. The AAUT consists of 17 items, of which we used 14 that involved purposeful UE movements.

#### Measuring impairment and performance of more-affected arm

With the IMES, impairment was assessed by the average movement duration of the more-affected arm in seconds (log-transformed) during the forced-choice block of the no-time constraint condition. Performance was measured as the total number of successful reaches with the more-affected arm during the forced-choice block in the fast-time constraint condition. To present this measurement as a percentage, the count was divided by the total number of targets (45 targets) in the forced-choice block. For the clinical tests, the Fugl-Meyer Assessment for Upper Extremity (FMA-UE) and the Wolf Motor Function Test with time domain (WMFT-time) were used to measure the impairment and performance of the more- affected arm, respectively. We used only the eight (of 17) items in the WMFT that were relevant to the reaching movement. The sum of each item on the FMA and the average time of the eight WMFT items (log-transformed) were calculated.

#### Effects of autonomy support on behavior changes

To understand how the participants changed their behaviors during the training session using the IMES, we compared the early (first 2 training days) and late (last 2 training days) phases with respect to the TDP, total use, successful/failed experiences, success rate, and perseverance (how patients with stroke endured failure) of the more-affected arm.

The TDP was recorded in each IMES training block throughout the entire training session. The total use of the more-affected arm was calculated by dividing the number of targets used by the more-affected arm by the total number of targets (100 targets) in each training block and then representing it as a percentage. We further examined the total use of the more- affected arm in two categories: successful and failed experiences. Successful experience was measured by counting the number of targets successfully reached by participants in each IMES training block, whereas failed experience was determined as the opposite.

The success rate was defined as the number of successful uses of the more-affected arm over the total use (success + failure) of the more-affected arm and is represented as a percentage. To investigate the influence of reinforcement history on arm choice, the perseverance of how much the participants with stroke endured failures was measured. Perseverance was calculated as the total use of the more-affected arm in the current trial *t*, even though the participants with stroke missed the target using the same arm in the previous trial *t-1*. A higher perseverance value indicated that the participants were less sensitive to failure.

#### Measuring self-efficacy for using more-affected arm

A question for measuring self-efficacy was developed with reference to Confidence in the Arm and Hand Movement (CAHM), a questionnaire that asks the individual to rate the level of confidence performing functional tasks using the UE [22]. Because self-efficacy is task-specific, we modified it to fit the IMES. After each training session, the participants were asked “How confident are you when catching an animal character using the more-affected arm?” and they answered on a scale of 0–100 on a scoring board, where 0 indicates very uncertain/unconfident and 100 indicates very certain/confident. The data obtained from the first and last training days (Days 2 and 10) were averaged for further analysis.

### Blinding

An independent evaluator blinded to participant recruitment, data collection, and group allocation analyzed all outcomes. The participants did not know which group they were in and were not allowed to discuss their training with each other.

### Statistical analysis

All statistical analyses were performed using R Studio 1.2, and the significance level was set at p < 0.05. The demographic information between groups was compared using either the Wilcoxon Ranked-Sum Test or the t-test, depending on the data distribution. A mixed-effect linear regression analysis was used to determine the impact of an individualized IMES training program on clinical and motivational variables. Test (pre vs. post) and group (autonomy support vs. control) were set as categorical variables of the fixed effect, and each individual was set as a random intercept. Four different analysis models were constructed as follows. The first two models included only one factor (e.g., Group or Test), while the last two models had two factors with and without interaction terms (e.g., Group + Test for model 3, Group × Test for model 4). The *anova* function in R studio was applied for model comparison and to define the best-fit model with the lowest Akaike information criterion. Once the best-fit model was selected, the model diagnostics, including visual inspection of outliers via the qq-norm plot, calculated Cohen’s distance. If necessary, Tukey’s post-hoc test was conducted. The effect size, measured as omega-squared (ω2), was calculated. We used phase (early vs. late) instead of test (pre vs. post) when analyzing participants’ autonomy-supporting behavior during training. To investigate whether changes in successful experiences during training sessions were associated with changes in IMES and clinical and motivational variables, correlation analyses were conducted using Spearman or Pearson methods depending on the data distribution. 

## Results

### Participants

Twenty-six individuals with mild to moderate motor impairment in the subacute or chronic stroke phase participated in this study (Table 1). The autonomy support and control groups did not differ in age or FMA, MMSE, and Edinburgh test results (all p > 0.05). All participants were at least 6 months after stroke onset except for two in the control group (C7 and C12, < 3 months). The time after stroke onset did not differ significantly between groups (p = 0.08), but the control group had a relatively shorter mean onset time than the autonomy support group.

### Changes in IMES and clinical variables before versus after training

#### Training effects on the use of the more-affected arm

**Fig. 3:**
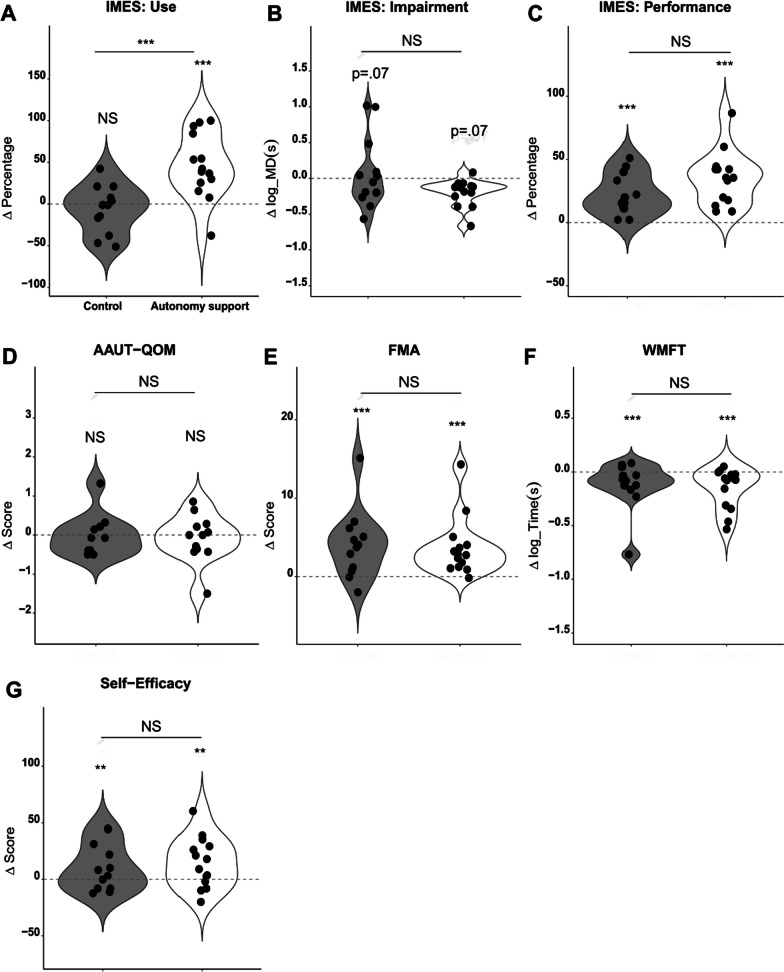
The group comparison between the autonomy support and control groups after the training. Each participant was represented by a dot, and the gray and white violin plots represented the control and autonomy support groups, respectively. The change in the use of the more-affected arm, measured in the free-choice block of the IMES (**A**), was higher in the autonomy support group after the training. However, there were no significant differences in the change in AAUT-QOM (**D**) between the pre- and post-training tests or between the control and autonomy support groups. Significant differences were found in performance, measured in the forced-choice block of the IMES (**C**), FMA (**E**), WMFT (**F**), self-efficacy (**G**), and the marginal difference was found in the movement duration (**B**), between the pre-training and post-training tests. However, no significant group differences were observed in these variables. *NS* non-significant, *p < 0.05, **p < 0.01, ***p < 0.001

The use of the more-affected measured by the IMES test session showed an interaction between the Group and Test variables. Post-hoc test revealed that the use of the more-affected arm did not improve in the control group, even after the training sessions (t = 0.612, p = 0.927) (Fig. 3A). However, the autonomy support group showed a significant increase in the use of the more-affected arm after training (t = − 4.966, p < 0.001). Neither group differed in their use in the pre-test (t = 1.471, p = 0.463) but significantly differed in the post-test (t = − 3.315, p = 0.009). However, the use measured by the AAUT-QOM score did not change after training (t = − 0.207, p = 0.836), and the scores did not differ significantly between groups (t = − 0.754, p = 0.451; Fig. 3D).

#### Training effects on the impairment, performance, and self-efficacy of the more-affected arm

The impairment measured by the IMES (e.g., average movement duration) was enhanced after the training sessions, but it did not reach the significance for both groups (t = − 1.782, p = 0.07, effect size = 0.07). Performance, which was measured in the forced-choice block of the fast-time constraint condition in the IMES, was significantly higher in the post-training test than pre-training test in both groups (t = 7.562, p < 0.0001, effect size = 0.68). However, there were no intergroup differences in impairment (t = − 0.981, p = 0.326) or performance (t = 0.621, p = 0.535; Fig. 3B, C).

We performed the same comparison for impairment and performance but with the clinical outcome measures (Table 2; Fig. 3). The total FMA scores increased after training in both groups (t = 5.047, p < 0.0001, effect size = 0.48), while log-transformed WMFT—time values significantly decreased after training in both groups (t = − 3.418, p < 0.001, effect size = 0.28). However, the control and autonomy support groups did not differ in terms of FMA (t = − 0.719, p = 0.472) or WMFT time (t = 0.716, p = 0.474; Fig. 3E, F). Self-efficacy increased from the pre-training to post-training test in both groups (t = 3.029, p = 0.002, effect size = 0.23; Table 2). However, these changes did not differ across groups (t = 0.091, p = 0.927; Figs. 3G; Table 2).

**Table 2:** IMES, clinical, and motivation variables before and after training

	Control			Autonomy support			Group p
	Pre	Post	P	Pre	Post	P	
IMES variables							
Use	36.85 ± 31.29	30.74 ± 17.97	0.927	20.87 ± 28.17	66.74 ± 30.31	< 0.001***	< 0.001***
Impairment	0.62 ± 0.27^a^	0.61 ± 0.3	P = 0.07	0.64 ± 0.23	0.48 ± 0.13	P = 0.07	0.326
Performance	39.44 ± 26.87	62.03 ± 30.23	< 0.001***	39.52 ± 26.73	74.60 ± 26.68	< 0.001***	0.535
Clinical variables							
AAUT-QOM	2.21 ± 1.2	2.30 ± 1.21	0.836	1.9 ± 1.53	1.86 ± 1.39	0.836	0.451
FMA-UE	45.92 ± 12.24	50 ± 10.57	< 0.001***	42.00 ± 17.28	45.64 ± 16.86	< 0.001***	0.472
WMFT	0.63 ± 0.21	0.51 ± 0.11	< 0.001***	0.71 ± 0.34	0.56 ± 0.20	< 0.001***	0.474
Motivation variables							
Self-efficacy	57.10 ± 20.21	67.38 ± 26.77	0.002**	55.76 ± 28.45	70.34 ± 23.47	0.002**	0.927

^a^Mean ± Standard Deviation, *p < 0.05, **p < 0.01, ***p < 0.001

Impairment in IMES variables was represented as a log-transformed movement duration and performance and use in IMES variables were represented as a percentage. AAUT-QOM, Actual Amount of Use Test with Quality of Movement scale; FMA-UE, Fugl-Meyer Assessment for Upper Extremity; WMFT, Wolf Motor Function Test; pre, pre-training test; post, post-training test. P denotes the significance level for comparisons between pre- and post-training within each group. Group p indicates the significance level when comparing pre-and post-training changes between the control and autonomy support groups

### Participant behavior changes by autonomy support during training

**Fig. 4:**
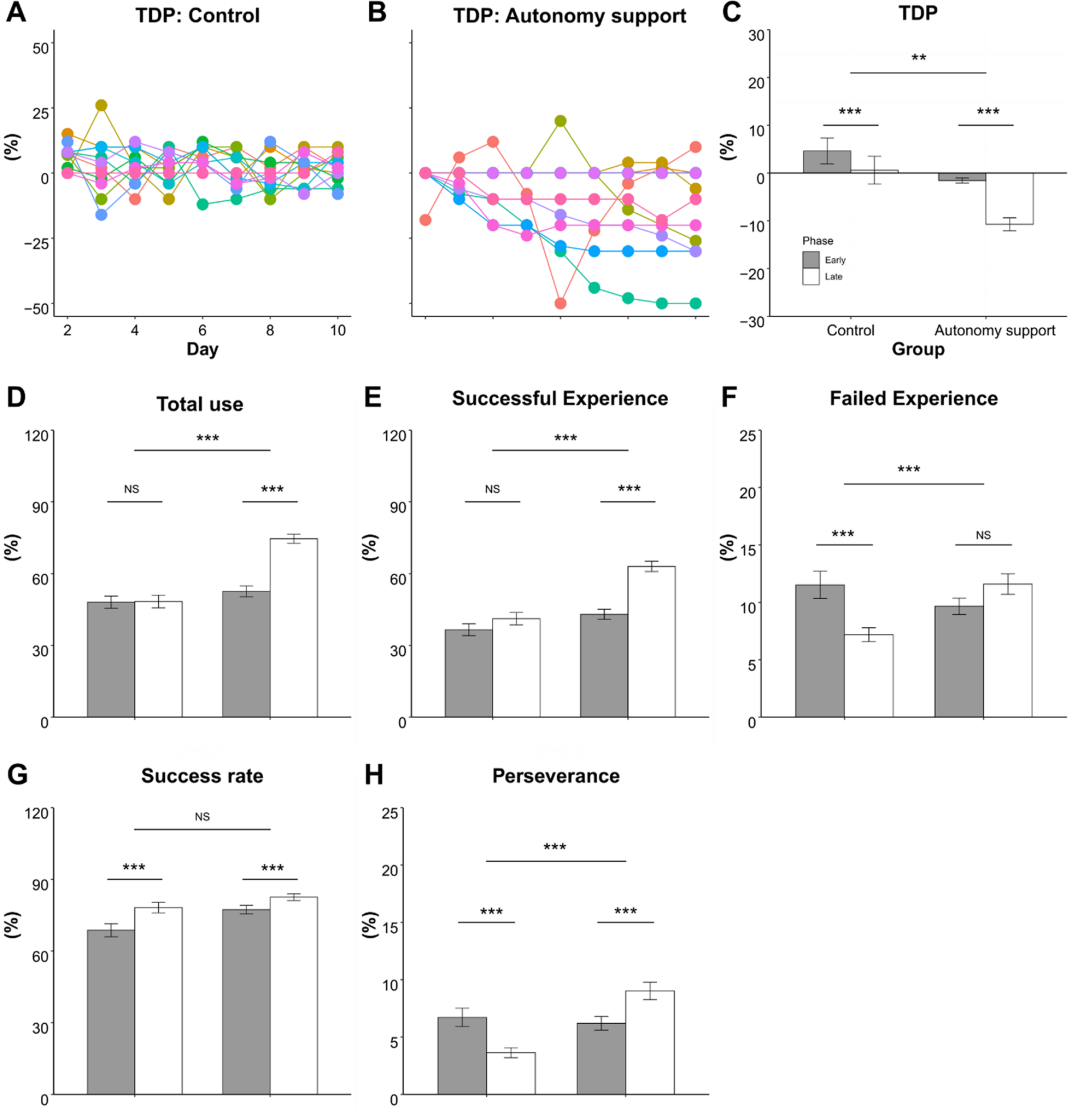
The task difficulty parameter (TDP) for the time limit and successful experience during training sessions. The individual TDP values across training sessions were plotted for both the control group (**A**) and the autonomy support group (**B**). The averaged TDP, total use, and successful experience during the early and late phases of training were presented (**C**–**E**). The failed experience (**F**), the success rate (**G**) and the perseverance (**H**) during the early and late phases were shown. It was observed that the TDP was lower in the autonomy support group compared to the control group. Additionally, the autonomy support group had significantly higher levels of successful experience compared to the control group. *NS* non-significant, **p < 0.01, ***p < 0.001

Figure 4A, B represent changes in the TDP for the time limit across training sessions. As expected, the TDP of the control group was randomized across training sessions. However, each participant in the autonomy support group selected a different TDP over time. Both groups showed decreases in the TDP (i.e., increased task difficulty) from the early to late phases (t = − 3.332, p = 0.001, effect size = 0.02; Fig. 4C). However, the TDP of the autonomy support group was always lower than that of the control group (t = − 3.22, p = 0.01, effect size = 0.28).

As noted above, the primary outcome—use of the more-affected arm—increased in the autonomy support group after training (Fig. 3C). This trend was observed even during the training sessions. Total use of the more-affected arm, including either success or failure, increased in the late versus early phase for the autonomy support group (p < 0.0001; Fig. 4D). However, the control group did not (p = 0.342). We also investigated other factors during the training sessions. The total use was further divided into  successful experiences and failed experiences. Successful experience did not change in the control group (t = − 2.186, p = 0.129), but it increased in the autonomy support group (t = − 10.21, p < 0.0001, effect size with omega = 0.10; Fig. 4E). While failed experiences (failure when using the more-affected arm) significantly decreased in the control group (t = 4.846, p < 0.0001), they marginally increased in the autonomy support group (t = − 2.508, p = 0.059; Fig. 4F). Success rates increased during late training in both groups (t = 15.503, p < 0.0001) but did not differ between groups (t = 1.02, p = 0.306; Fig. 4G). Finally, perseverance decreased in the control group (t = 4.103, p = 0.003) but increased in the autonomy support group (t = − 4.106, p = 0.003) from the early to the late training phases (Fig. 4H).

### Correlation between changes in successful experience during training and changes in IMES, clinical, and motivation variables before versus after training

**Fig. 5:**
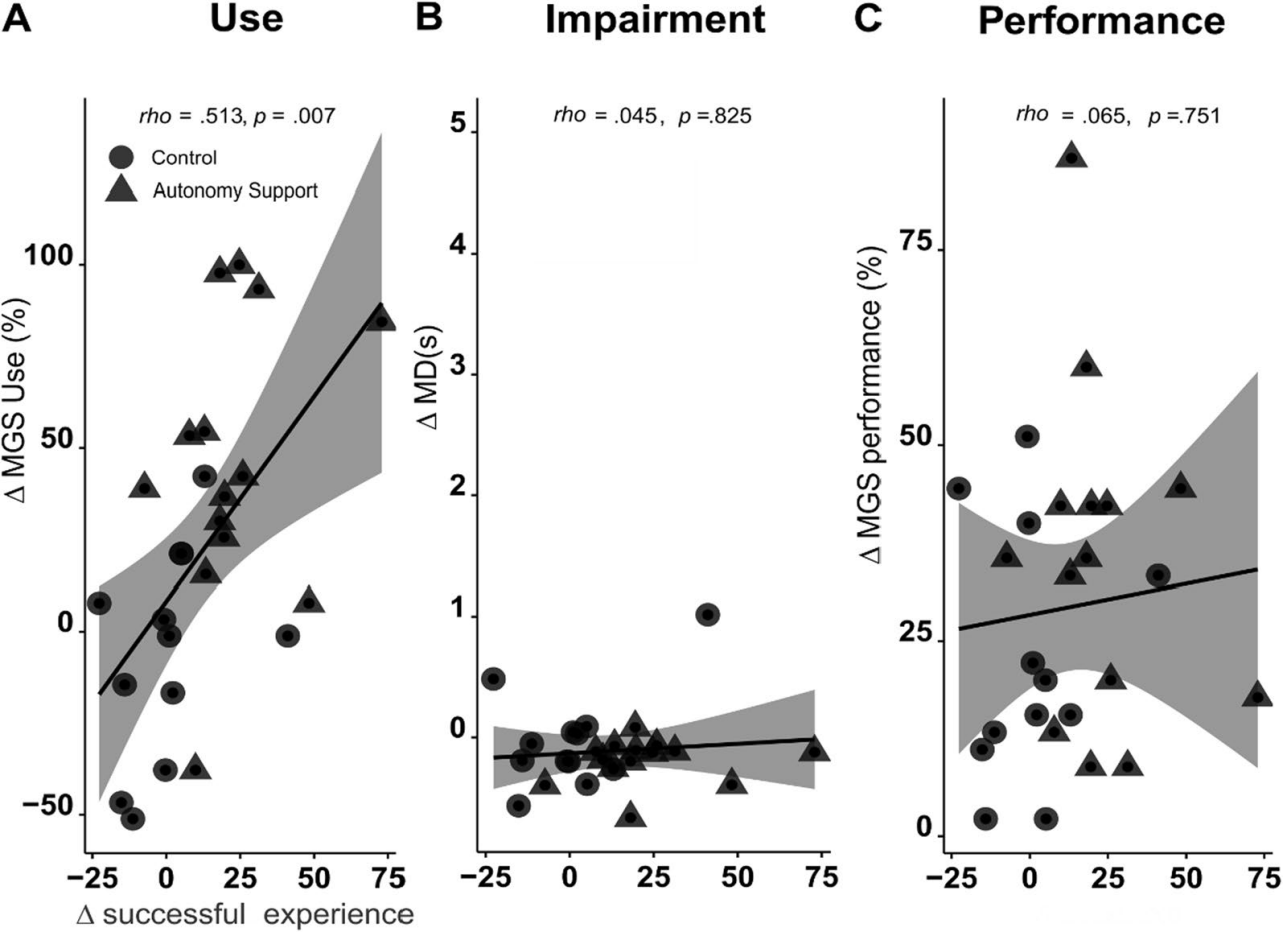
Correlations between changes in successful experience and variables in the IMES. **A** There was a significant correlation between changes in the use of the more-affected arm and successful experience (p = 0.007). However, Impairment (**B**) and performance (**C**) did not show significant correlations with successful experience. Each data point represents an individual from both the control and autonomy support groups

In the analysis of behavioral changes during training, the successful experience significantly changed in the late phase of training only for the autonomy support group. Thus, we investigated whether successful experience during training affects the use, impairment, and performance of the more-affected arm. Specifically, we performed a correlation analysis of changes in the successful experience between the early and late phases of the training sessions and changes in use, impairment, and performance before versus after training. We found a significant correlation between changes in the use of the more-affected arm measured by the IMES and changes in successful experiences (Spearman correlation, rho = 0.513 p = 0.007; Fig. 5A). However, changes in impairment and performance did not correlate with changes in successful experiences (all, p > 0.05) (Fig. 5B, C).

We additionally tested their association with motivational variables (i.e., self-efficacy). However, none of the others measured by the IMES and clinical tests correlated with self-efficacy (all, p > 0.05).

## Discussion

Our results can be summarized in three aspects: (1) The autonomy support group selected more difficult tasks across training sessions (i.e., the TDP significantly decreased over the training blocks; Fig. 4C); (2) Both groups showed improved performance (performance in IMES, and WMFT in clinical tests; Fig. 3B, E) and motivation (self-efficacy; Fig. 3G) with no group differences; (3) However, only the participants in the autonomy support group showed increased successful experiences during the training session (Fig. 4E) and increased use of the more-affected arm (Fig. 3C) measured by the IMES test session after training compared to those in the control group. The two measures (successful experience and use of the more-affected arm) were moderately associated (Fig. 5C); in other words, a more successful experience during the training period was associated with more use after the training. Note that the change in the use measured by the AAUT did not reach a significant level and we discussed this issue later.

### Role of autonomy support in the use of the more-affected arm

In our experiment, the autonomy support allowing participants with stroke to modulate task difficulty provided more opportunities to experience successful use of the more-affected arm. This successful experience might increase the use of the more-affected arm measured by the IMES, as other researchers have suggested that successful experiences are critical ingredients in enhancing recovery [25, 36–38]. Owing to the instruction and training environment related to autonomy support, patients in the autonomy support group might be aware of the training goal, such as using the more- affected arm as much as possible. Awareness of this task goal eventually might affect the participants’ decision to select one arm over the other.

Interestingly, the participants in the autonomy support group selected a shorter time limit (decreased TDP; Fig. 4B, C). This may be because completing challenging tasks results in greater internal rewards than completing easy tasks [39]. In this case, it is crucial that the participants successfully complete a challenging task and not just engage in a challenging task [40]. Thus, successful experiences during the current training block in our study might increase satisfaction and competence in the reaching task, leading to the selection of the more challenging task for the next training block. Based on these results, we suggest that IT device–based systems for rehabilitation (e.g., rehabilitation robots) should be incorporated into a rehabilitation framework that provides autonomy support and reduces the load on clinicians. Autonomy support may help prevent patients with stroke from “slacking,” a situation in which the patient is not actively participating in the therapy session or not putting in the required effort, energy consumption, and attention [41].

### Selective influence of autonomy support on recovery of arm use after stroke

Literature on motor learning reveals that giving learners control over certain practice conditions enhances their motor skill learning [42]. In our study, highly repetitive reaching movements without autonomy support improved motor performance, but these gains were not transferred to using the more-affected arm. Conversely, the autonomy support group demonstrated improved performance and use of the more-affected arm. This result is in line with a previous study in which participants who received intervention involving autonomy support showed increased use of the more-affected arm than the control group immediately after training. However, their motor impairment and performance improved in both intervention and control groups without intergroup differences [15, 43]. Although this group difference in use disappeared by the 6-month follow-up test, the participants with intervention involving autonomy support had the benefit of improving faster than those in the control group [15]. Together, our results provide evidence that autonomy support selectively affects the use of the more-affected arm, while training itself affects its performance.

### Relationship between autonomy support and self-efficacy

Here we investigated the belief that confidence level is the key to the use of the more-affected arm, as confidence level was measured by self-efficacy. However, our study found no association between the use of the more-affected arm and self-efficacy. Although the control and autonomy support groups showed enhanced self-efficacy after training, they did not differ between the two groups despite different training protocols (Fig. 3G). However, as noted above, the more-affected arm was used significantly more frequently in the autonomy support versus control group after training (Fig. 3C). Correlation analyses revealed that changes in self-efficacy were not correlated with changes in use of the more-affected arm (Spearman correlation, rho = 0.446, p = 0.101, data not shown).

Our findings contradict those of the previous studies suggesting that self-efficacy is linked to more-affected UE selection; the higher the self-efficacy, the greater the use of that arm [12–14]. If this was applied to our study, because self-efficacy increased in both groups, the use of the more-affected arm should have increased in both groups. However, this was not the case in the present study. To explain this discrepancy, we needed to closely examine success rates, failure experience, and perseverance. Even though the success rates increased after the training sessions, they did not differ between groups (Fig. 4G), as self-efficacy did (Fig. 3G). The participants in the two groups showed different strategies for improving their success rates. Specifically, those in the control group performed conservative training. They did not explore or extend the use of the more-affected arm; instead, they tried to diminish the experience of failure (Fig. 3E). Thus, the overall success rate increased, but remained the same successful experiences in the early and late training phases. Conversely, participants in the autonomy support group became less sensitive to failure over time. Their perseverance (e.g., continued use of the more-affected arm even after failure) increased, while that of the control group decreased. As a result, the participants in the autonomy support group had more successful experiences using the more-affected arm during training(Fig. 4E), which might have increased the use of that arm.

### Discrepancy between IMES and clinical test results

In our study, we observed an increase in the use of the more-affected arm with autonomy support only as measured by IMES (Fig. 3A). However, we did not identify any group or training effects on the use of the more-affected arm when measuring the AAUT (Fig. 3D). This may be due to the nature of task-specific motor learning. Unlike previous studies in which participants were trained with task-oriented practice emphasizing real-world activities [7, 15], we used a simple reaching task during training; thus, the gains in IMES did not extend to the clinical test. Nevertheless, one can confirm that even with a simple reaching task with an IT device, autonomy support is practical for performance enhancement and the use of the more-affected UE by post-stroke patients. Further studies with IT devices involving autonomy support and training for instrumental use of the more-affected arm may show a distinguishable effect of autonomy support on real-world arm choices.

## Limitations

This study had several limitations. First, only a small number of participants were included in each group, so our data was interpreted cautiously. Second, the control group was subjected to random task difficulty levels, which may have hindered learning in the use of the more-affected arm. In fact, the IMES system incorporates a 'personalized training mode' that dynamically adjusts task difficulty based on the use of the more-affected arm in each trial. This mode delivers an intermediate level of task difficulty, which is recognized as optimal for promoting both motivation and learning. However, we intentionally opted not to use this mode to eliminate any factors that could artificially enhance motivation within the control group. Future studies should investigate the comparison between the personalized training mode and autonomy support training. Lastly, people commonly use the arm and hands in a bimanual fashion; often, each arm and hand have different roles in certain tasks (e.g., writing a note, writing with the right hand, and holding the paper with the left hand). However, we did not train our participants with stroke via symmetrical or asymmetrical bimanual movements. Furthermore, our training was a reaching task, which was different from the typical real-world use of arm and hand movements (e.g., reaching and manipulating objects). A larger sample size with imaging data and a comparison of treatment protocols between forced- and free-choice training are also needed for future research.

## Conclusions

When the participants with stroke had an opportunity for autonomy support by actively participating to modify the task difficulty, they were successful in using the more-affected arm, which may have influenced the increased use of that arm after stroke. This successful experience during the training phase is required for patients to understand the importance and benefits of using the more-affected arm. With high repetition and autonomy support, they may be able to use the arm more habitually. In addition, an IT device combined with autonomy support is practical for performance enhancement and the use of more-affected arm in post-stroke. Thus, developing IT devices involving autonomy support and instrumental use of the more-affected arm will reveal a distinguishable effect of autonomy support on real-world arm choices.

## Data Availability

The data is available upon request.

## Abbreviations

AAUT: Actual amount of use test
FMA: Fugl-Meyer Assessment
IMES: Individualized Motivation Enhancement System
IT: Information technology
MMSE: Mini Mental State Examination
QOM: Quality of movement
TDP: Task difficulty parameter
UE: Upper extremity
WMFT: Wolf Motor Function Test
