# Correction: Autonomy support encourages use of more-afected arm post-stroke

**DOI:** 10.1186/s12984-023-01244-2

**Published:** 2023-09-26

**Authors:** Sujin Kim, Yumi Shin, Yeonwoo Jeong, Seungyoung Na, Cheol E. Han

**Affiliations:** 1 Department of Physical Therapy, Jeonju University, Jeonju, South Korea; 2 Department of Rehabilitative and Assistive Technology, National Rehabilitation Center, Seoul, South Korea; 3 Department of Rehabilitation and Medicine, Ongoul Rehabilitation Hospital, Jeonju, South Korea; 4 Department of Electronics and Information Engineering, Korea University, Sejong, South Korea; 5 Interdisciplinary Graduate Program for Artificial Intelligence Smart Convergence Technology, Korea University, Sejong, South Korea

Following publication of the original article [1], in this article there is minor changes in the figures, tables and the same has been updated

The original article has been corrected.
